# THE APPLICATION OF MASS OBSERVATION DATA IN SOCIAL RESEARCH

**DOI:** 10.1093/geroni/igz038.2777

**Published:** 2019-11-08

**Authors:** Paul Kingston

**Affiliations:** University of Chester, Chester, United Kingdom

## Abstract

Responding to the opportunities and challenges of an ageing world the University of Chester established the Centre for Ageing, Mental Health and Veterans’ Studies in 2013 to provide research, consultancy and education, with the aim of promoting innovation in health and social care services for older people. This symposium brings together researchers from a wide range of disciplines and career stages, to explore the utility of Mass observation data in social research in the field of gerontology. The Mass Observation Project, established in 1937, documents the lives of ordinary people living in the UK, and explores a wide range of social issues. The symposium comprises four separate papers. The Methodological Relevance of Mass Observation Data: This preliminary overview will outline the mass observation archive, highlighting challenges and issues encountered utilising the data produced in social research. Personal Narratives of Ageing: This paper presents personal narratives reflecting on the ageing process, and growing older in the UK. The Health Impact of Scams: This presentation will offer new and alternate insights into ‘scams’ and the health effects of fraud on older people, using data from the mass observation directive commissioned by the centre. Perceptions of Dementia: This paper presents a perspective on the public knowledge and understanding about dementia not previously considered, where respondents have written openly about their own experiences, and reflected on their perception of the wider public’s knowledge and understanding about dementia.

